# Hybrid PET/optical imaging of integrin α_V_β_3_ receptor expression using a ^64^Cu-labeled streptavidin/biotin-based dimeric RGD peptide

**DOI:** 10.1186/s13550-015-0140-0

**Published:** 2015-10-31

**Authors:** Choong Mo Kang, Hyun-Jung Koo, Gwang Il An, Yearn Seong Choe, Joon Young Choi, Kyung-Han Lee, Byung-Tae Kim

**Affiliations:** Department of Nuclear Medicine, Samsung Medical Center, Sungkyunkwan University School of Medicine, Seoul, 06351 Korea; Department of Health Sciences and Technology, SAIHST, Sungkyunkwan University, Seoul, 06351 Korea; Molecular Imaging Research Center, Korea Institute of Radiological and Medical Sciences, Seoul, 01812 Korea

**Keywords:** Streptavidin/biotin, Dimeric RGD peptide, ^64^Cu, Integrin α_V_β_3_, Hybrid PET/optical imaging

## Abstract

**Background:**

Hybrid PET/optical imaging provides quantitative and complementary information for diagnosis of tumors. Herein, we developed a ^64^Cu-labeled AlexaFluor 680-streptavidin ((AF)SAv)/biotin-based dimeric cyclic RGD peptide (RGD_2_) for hybrid PET/optical imaging of integrin α_V_β_3_ expression.

**Methods:**

^64^Cu-1,4,7,10-tetraazacyclododecane-*N*,*N*′,*N*′′,*N*′′′-tetraacetic acid (DOTA)-(AF)SAv/biotin-PEG-RGD_2_ was prepared by formation of a complex comprising DOTA-(AF)SAv and biotin-PEG-RGD_2_, followed by radiolabeling with ^64^Cu. Receptor binding studies of DOTA-(AF)SAv/biotin-PEG-RGD_2_ were performed using U87MG cells and ^125^I-RGDyK as the radioligand, and cellular uptake studies of ^64^Cu-DOTA-(AF)SAv/biotin-PEG-RGD_2_ were also performed. MicroPET imaging followed by optical imaging of U87MG tumor-bearing mice was acquired after injection of the hybrid probe, and region of interest (ROI) analysis of tumors was performed. Ex vivo PET/optical imaging and biodistribution studies of the major tissues were performed after the in vivo imaging, and immunofluorescence staining of the tumor tissue sections was carried out.

**Results:**

^64^Cu-DOTA-(AF)SAv/biotin-PEG-RGD_2_ was prepared in 52.1 ± 5.4 % radiochemical yield and with specific activity of 1.0 ± 0.1 GBq/mg. Receptor binding studies showed that DOTA-(AF)SAv/biotin-PEG-RGD_2_ had higher binding affinity for integrin α_V_β_3_ than RGD_2_, reflecting a possible polyvalency effect. Moreover, the hybrid probe revealed time-dependent uptake by U87MG cells. In a microPET/optical imaging study, the hybrid probe demonstrated high accumulation in tumors; ROI analysis revealed 2.7 ± 0.2 % ID/g at 1 h and 4.7 ± 0.2 % ID/g at 21 h after injection, and subsequently acquired optical images showed tumors with strong fluorescence intensity. Ex vivo PET/optical images of the major tissues confirmed the in vivo imaging data, and biodistribution studies demonstrated high and specific uptake in tumors (4.8 ± 0.1 % ID/g). Immunofluorescence staining showed the formation of new blood vessels in tumor tissues, suggesting that the tumor uptake was due to specific binding of the hybrid probe to integrin α_V_β_3_ expressed on tumor cells.

**Conclusions:**

These results indicate that a ^64^Cu-DOTA-(AF)SAv/biotin-PEG-RGD_2_ is able to provide quantitative information on hybrid PET/optical imaging of integrin α_V_β_3_ expression.

## Background

Demand for hybrid imaging probes has been growing with advances in hybrid imaging systems such as PET/CT, SPECT/CT, and PET/MRI, and expansion of their clinical uses [1]. PET provides sensitive and quantitative information, and moreover, PET probes are easily translated for clinical use compared with probes used for other modalities [2]. However, PET has the disadvantage of low spatial resolution. MRI offers the highest spatial resolution but has low sensitivity [2]. Optical imaging has the advantages of real-time imaging with high sensitivity and can potentially be used in intraoperative image-guided surgery [2, 3]. However, it is mainly limited to small animal imaging due to low depth penetration. Therefore, hybrid imaging, by compensating for the drawbacks of single imaging modalities, is more accurate and allows for earlier diagnosis of diseases. This has spurred the development of hybrid imaging probes containing diverse functionalities for application in hybrid PET/optical, PET/MR, and other combinations of imaging modalities.

To develop hybrid imaging probes, nanoparticles are commonly used as platforms because of the ease of addition of diverse functional groups including tumor-targeting compounds. For tumor angiogenesis targeting, cyclic Arg-Gly-Asp (RGD) peptides, selective antagonists of integrin α_V_β_3_, have been radiolabeled with various radioisotopes. The most well-characterized ligand, ^18^F-labeled galacto-RGD peptide, has shown promise for tumor imaging of melanoma M21 tumor-bearing mice and diagnosis of cancer patients [4, 5]. With its longer half-life, ^64^Cu-labeled RGD peptide showed prolonged tumor uptake in MDA-MB-435 tumor-bearing mice [6]. In addition, PEGylation of RGD peptides improved in vivo pharmacokinetics; ^64^Cu-labeled PEGylated RGD peptide accumulated in tumors at early time points and rapidly washed out from the blood of U87MG tumor-bearing mice compared with ^64^Cu-labeled RGD peptide [7, 8]. Multimeric RGD peptides, such as ^64^Cu-labeled RGD dimer and tetramer, were shown to possess not only higher integrin binding affinity compared with the corresponding monomer but also prolonged tumor retention and increased renal excretion in tumor-bearing mice, which were possibly attributed to a polyvalency effect [9, 10]. Other dimeric RGD peptides, labeled with ^18^F, ^68^Ga, and ^99m^Tc, have also been shown to have superior in vivo properties than their corresponding monomers [11–14].

Nanoparticles such as liposomes, quantum dots, gold nanoparticles, carbon nanotubes, and others have been conjugated with RGD peptides and utilized for hybrid imaging or improved tumor targeting. ^111^In-DTPA-labeled liposomes conjugated with RGD peptide were designed for hybrid SPECT/MR imaging of integrin α_V_β_3_. Although ^111^In-labeled liposomes exhibited low tumor uptake in U87MG tumor-bearing mice, Gd-labeled liposomes were taken up by the tumors of both U87MG and M21 tumor-bearing mice, based on R1 values [15]. ^64^Cu-labeled quantum dots (Qdots) modified with RGD peptides were developed for PET and near-infrared fluorescence (NIRF) imaging, and the tumor vasculature was visualized by both PET and ex vivo NIRF imaging [16]. In another study, RGD peptides and DOTA were conjugated to polyaspartic acid-coated iron oxide nanoparticles, which were then labeled with ^64^Cu. Hybrid PET/MR imaging of the particles in U87MG tumor-bearing mice showed integrin α_V_β_3_ expression in the tumor [17]. ^125^I-labeled RGD-PEG-gold nanoparticles were prepared by conjugating PEG to the gold particles and RGD peptide to PEG, and then finally radiolabeling the particles with ^125^I. RGD-PEG-gold nanoparticles exhibited much higher binding affinity for integrin α_V_β_3_ on U87MG cells than free RGDyC peptide. SPECT/CT images of U87MG tumor-bearing mice showed fast tumor uptake of the gold particles [18]. Single-walled carbon nanotubes have also been used as a platform for tumor angiogenesis imaging; carbon nanotubes coated with RGD- and DOTA-conjugated PEG-phospholipids were labeled with ^64^Cu, and then the resulting nanotubes had a high tumor uptake in U87MG tumor-bearing mice. Ex vivo Raman spectroscopy data of the major tissues confirmed the microPET imaging data [19].

Previously, we and others showed that a streptavidin (SAv) and biotin complex can serve as a useful platform for the development of hybrid imaging probes due to the strong interaction between SAv and biotin, with a dissociation constant of approximately 10^−14^ M [20]. Examples of these probes include ^99m^Tc-HYNIC-labeled quantum dots (Qdots)-SAv/biotin-PEG-EGF, SAv/three biotinylated ^111^In-DOTA, Cy5.5 and anti-Her2 antibody, and ^64^Cu-DOTA-(AF)SAv/biotin-PEG-VEGF_121_ [21–23]. Unlike most nanoparticles, antibody-conjugated SAv and ^90^Y-DOTA-biotin have been used clinically for pretargeted radioimmunotherapy, which was shown to be safe [24].

In this study, we extended application of the SAv/biotin platform to the development of an E[cyclic(RGDyK)]_2_ (RGD_2_)-based hybrid imaging probe for tumor angiogenesis imaging. Therefore, ^64^Cu-DOTA-(AlexaFluor680; AF)SAv/biotin-PEG-RGD_2_ was prepared and evaluated for hybrid PET/optical imaging of integrin α_V_β_3_ receptor expression in U87MG tumor-bearing mice.

## Methods

### Materials and equipment

RGD_2_ was purchased from Peptide International (Louisville, KY, USA), biotin-PEG(3400)-NHS ester was from Nanocs (New York, NY, USA), (AF)SAv was from Life Technologies (Carlsbad, CA, USA), and DOTA-NHS ester was from Macrocyclics (Dallas, TX, USA). ^64^CuCl_2_ was kindly provided by KIRAMS (Seoul, Korea). PD-10 columns were purchased from Amersham Biosciences (Piscataway, NJ, USA), Amicon filters were from Millipore (Billerica, MA, USA), and spin columns were from Thermo Scientific (Rockford, IL, USA). Chelex 100 resin (50–100 mesh) and other chemicals were purchased from Sigma-Aldrich (St. Louis, MO, USA), and BCA protein assay kits were from Pierce (Rockford, IL, USA). All buffers used for synthesis and radiolabeling were pretreated with Chelex 100 resin to ensure that they were metal-free. Matrix-assisted laser desorption ionization time of flight (MALDI-TOF) mass spectrometry was performed on a Voyager-DETM STR Biospectrometry Workstation (Applied Biosystems, Foster City, CA, USA). Purification and analysis of products were performed by HPLC (Thermo Scientific, Waltham, MA, USA), and eluates were monitored using a UV detector (218 nm).

Radioactivity was measured using a dose calibrator (Biodex Medical Systems, Shirley, NY, USA), and tissue radioactivity was counted using an automatic gamma counter (PerkinElmer, Waltham, MA, USA). MicroPET and optical images were acquired at the Center for Molecular and Cellular Imaging, Samsung Biomedical Research Institute (SBRI, Seoul, Korea) using an Inveon microPET/CT scanner (Siemens Medical Solutions, Malvern, PA, USA) and a Xenogen IVIS Spectrum (Caliper Life Sciences, Hopkinton, MA, USA), respectively.

### Preparation of DOTA-(AF)SAv

(AF)SAv (1.3 mg) and 20 equivalents of DOTA-NHS ester (400 μg, 481.9 nmol) were dissolved in 800 μL of 0.1 M sodium carbonate buffer (pH 8.5), which was then stirred at room temperature for 18 h in the dark. At the end of the reaction, the reaction mixture was purified using a PD-10 column, concentrated using an Amicon filter (cut-off: 10 kDa), and then lyophilized. DOTA-(AF)SAv was obtained at 97.3 % yield, quantified using a BCA protein assay kit, and then analyzed by MALDI-TOF mass spectrometry.

### Preparation of biotin-PEG-RGD_2_

RGD_2_ (1.7 mg, 1.3 μmol) and biotin-PEG(3400)-NHS ester (2.4 mg, 0.6 μmol) were dissolved in 500 μL of 0.1 M sodium carbonate buffer (pH 8.5), which was then stirred at room temperature for 19 h. At the end of the reaction, the reaction mixture was purified by HPLC using a C18 column (YMC, 5 μm, 10 × 250 mm) with sequential programs using a mixture of 0.1 % trifluoroacetic acid (TFA) in water and CH_3_CN. The first isocratic program was a 90:10 mixture over 10 min, and the second gradient program was from a 90:10 mixture to a 35:65 mixture over 30 min. The flow rate was 3 mL/min. After lyophilization, biotin-PEG-RGD_2_ was analyzed by MALDI-TOF mass spectrometry.

### Preparation of ^64^Cu-DOTA-(AF)SAv/biotin-PEG-RGD_2_

Biotin-PEG-RGD_2_ (91.2 μg, 18.2 nmol) dissolved in acetate buffer (pH 6) was added to DOTA-(AF)SAv (201.9 μg, 3.6 nmol) in the same buffer. The reaction mixture (total volume of 200 μL) was stirred at room temperature for 1 h, and then incubated with ^64^CuCl_2_ (377 MBq) at 40 °C for 30 min with constant shaking. Unreacted ^64^CuCl_2_ and biotin-PEG-RGD_2_ in the reaction mixture were removed by centrifugation using a spin column (molecular weight (mw) cut-off 7 kDa).

In a separate experiment, an aliquot of DOTA-(AF)SAv/biotin-PEG-RGD_2_ was purified using a spin column (cut-off 7 kDa) and its purity was determined by HPLC using a Superdex™ 75 GL column (10 × 300 mm, GE Healthcare Life Sciences, Marlborough, MA, USA) and 0.01 M phosphate buffer as the eluent at a flow rate of 0.7 mL/min.

### Serum stability

^64^Cu-DOTA-(AF)SAv/biotin-PEG-RGD_2_ (98.4 MBq) in 0.01 M PBS (pH 7.4) was added to 50 % fetal bovine serum (FBS; Gibco, Brooklyn, NY, USA) and incubated at 37 °C for 0, 1, 3, 16, and 24 h. At the indicated time points, each sample was loaded onto a PD-10 column, and fractions were eluted using PBS (pH 7.4) and counted using a dose calibrator.

### Cell integrin receptor binding

The integrin α_V_β_3_ receptor binding study was performed using a previously reported procedure with a slight modification [25]. ^125^I-RGDyK was used as the radioligand and prepared immediately before use. U87MG cells (2 × 10^6^ cells/100 μL) cultured in minimum essential media (MEM; Gibco) were re-suspended in binding buffer (20 mM Tris–HCl buffer with 150 mM NaCl, 2 mM CaCl_2_, 1 mM MgCl_2_, 1 mM MnCl_2_, and 0.1 % bovine serum albumin (BSA); pH 7.4). ^125^I-RGDyK (2.7 kBq/80 μL of binding buffer) was added to tubes containing U87MG cells in the presence of different concentrations (0.1, 1, 10, 10^2^, 10^3^, 10^4^, and 10^5^ nM; 20 μL) of either DOTA-(AF)SAv/biotin-PEG-RGD_2_ or RGD_2_ at room temperature for 1 h. After incubation, the cells were washed three times with PBS for 5 min, re-suspended with PBS (1 mL), and then counted using a gamma counter. All experiments were performed in triplicate. IC_50_ values were determined using GraphPad Prism software 5.

### Cellular uptake

U87MG cells were cultured in MEM supplemented with 10 % FBS, streptomycin (100 μg/mL), and penicillin (100 units/mL). Cells were maintained at 37 °C in a humidified 5 % CO_2_ incubator. U87MG cells were seeded in 12-well plates at 5 × 10^5^ cells/well and cultured for 2 days. ^64^Cu-DOTA-(AF)SAv/biotin-PEG-RGD_2_ (111 kBq/2 μL) was added to each well, and the cells in a total volume of 0.5 mL were incubated at 37 °C for 1, 2, 16, and 21 h. After incubation, the cells were washed three times with PBS. Cell lysis was then carried out using 0.1 N NaOH, and the resulting lysate was counted using a gamma counter. For the blocking study, cells were incubated with the hybrid probe in the presence of RGD_2_ (10 μM) at 37 °C for 21 h and then treated as described above. All experiments were performed in triplicate.

### Ethics statement

This study was reviewed and approved by the Institutional Animal Care and Use Committee of SBRI. SBRI is an Association for Assessment and Accreditation of Laboratory Animal Care International accredited facility and abide by the Institute of Laboratory Animal Resources guide.

### In vivo microPET/optical imaging

U87MG tumor-bearing mice were prepared by subcutaneously inoculating U87MG cells (5 × 10^6^) into the right hind legs of 5-week-old BALB/c nude mice (male). When tumor size reached 407.4 ± 58.6 mm^3^ at 3 weeks after inoculation, the ^64^Cu-DOTA-(AF)SAv/biotin-PEG-RGD_2_ (6.9 ± 0.3 MBq/200 μL) was injected intravenously without (*n* = 3) or with RGD_2_ (20 mg/kg, *n* = 3) through the tail vein. MicroPET static images were acquired for 10 min at 1, 2, 16, and 21 h after injection. Immediately after microPET imaging, optical images of the mice were acquired (excitation: 675 nm, emission 720 nm) for 1 s.

The images obtained were reconstructed using three-dimensional ordered subset expectation maximization and then processed using Siemens Inveon Research Workplace 4.2. Regions of interest (ROIs) were drawn over tumors in the right legs, and the average signal levels in the ROIs were measured. Data are expressed as percent injected dose per gram of tissue (% ID/g). ROIs of optical images were drawn over tumors, and signal levels were measured using Living Image 3.2 software. These data are presented as photons per second per square centimeter per steradian (photons/s/cm^2^/sr).

### Ex vivo imaging and biodistribution studies

At the end of the in vivo microPET/optical imaging study, mice were sacrificed, and major tissues (heart, lungs, liver, spleen, kidneys, intestines, muscle, and tumor) were separated immediately and subjected to ex vivo imaging (microPET, 10-min static scan; optical imaging, 1-s exposure). ROIs of ex vivo images were drawn around each tissue, and the average signal level in the ROI was measured. For the biodistribution study, the tissues of interest and blood were then weighed and counted, and data are expressed as percent injected dose per gram of tissue.

### Immunofluorescence staining

Tumor tissues obtained after in vivo imaging were fixed in 4 % paraformaldehyde overnight. Specimens were then dehydrated in graded ethanol, embedded in paraffin, and sectioned at 5 μm on a Reichert microtome. The sections were blocked with 1 % BSA in PBS for 30 min and then incubated with goat anti-β_3_ (1:100; Santa Cruz Biotechnology, Santa Cruz, CA, USA) and rabbit anti-VEGFR2 antibodies (1:100; Santa Cruz Biotechnology) overnight at 4 °C. Subsequently, sections were stained with FITC-labeled anti-goat and rhodamine-labeled anti-rabbit antibodies (1:200; Santa Cruz Biotechnology). Hoechst 33342 (1 μg/mL; Cell Signaling Technology, Danvers, MA, USA) was used to stain the cell nuclei of tumor tissues. Stained tissue sections were examined under a microscope (×200; Nikon Eclipse 80i).

### Statistical analysis

Data were analyzed with unpaired, two-tailed Student’s *t* tests. Differences at the 95 % confidence level (*P* < 0.05) were considered statistically significant.

## Results

### Preparation of DOTA-(AF)SAv and biotin-PEG-RGD_2_

DOTA-(AF)SAv prepared from (AF)SAv and DOTA-NHS ester was readily purified using a size-exclusion column. MALDI-TOF mass spectrometric analysis indicated that the average number of DOTA molecules conjugated to (AF)SAv was 3.8 ± 0.6. In contrast, to prepare biotin-PEG-RGD_2_, excess amounts (2 equivalents) of RGD_2_ were used to consume as much biotin-PEG-NHS ester as possible, as this NHS ester eluted close to biotin-PEG-RGD_2_ on HPLC. The resulting biotin-PEG-RGD_2_ was purified by HPLC, but not using a size-exclusion column because of its similarity in molecular weight to the biotin-PEG-NHS ester. Biotin-PEG-RGD_2_ was obtained at 87.1 ± 8.1 % yield and identified by MALDI-TOF mass spectrometry: (m/z) [M + H]^+^ calcd. 5023.77, C_225_H_413_N_22_O_98_S; found 5024.57.

### Preparation of ^64^Cu-DOTA-(AF)SAv/biotin-PEG-RGD_2_

**Fig. 1:**
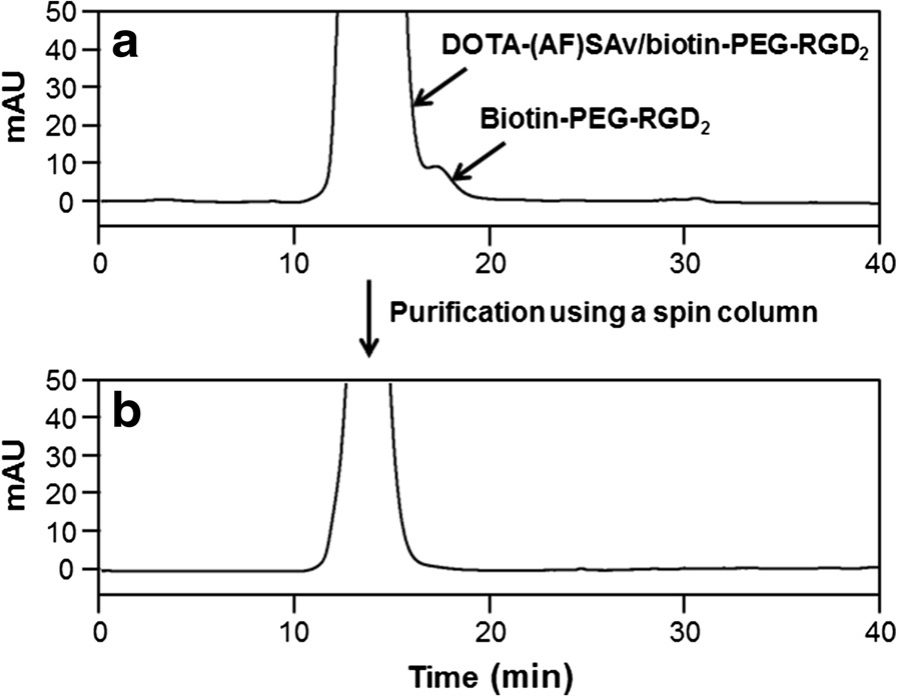
Size-exclusion HPLC profiles of DOTA-(AF)SAv/biotin-PEG-RGD_2_ before (**a**) and after purification (**b**) using a spin column

Purity of a DOTA-(AF)SAv/biotin-PEG-RGD_2_ complex after spin column purification was confirmed by HPLC using a Superdex™ 75 GL column (Fig. 1a, b). To reduce the requirement for two-step purification comprising DOTA-(AF)SAv/biotin-PEG-RGD_2_ purification using a spin column and ^64^Cu-DOTA-(AF)SAv/biotin-PEG-RGD_2_ purification using a PD-10 column, the hybrid probe was prepared in a two-step reaction and finally purified; DOTA-(AF)SAv and biotin-PEG-RGD_2_ were mixed at a ratio of 1 to 5, which was then labeled with ^64^Cu, and finally the hybrid probe was purified using a spin column. Non-decay-corrected radiochemical yield of the ^64^Cu-DOTA-(AF)SAv/biotin-PEG-RGD_2_ was 52.1 ± 5.4 % and its specific activity was 1.0 ± 0.1 GBq/mg.

### Serum stability

**Fig. 2:**
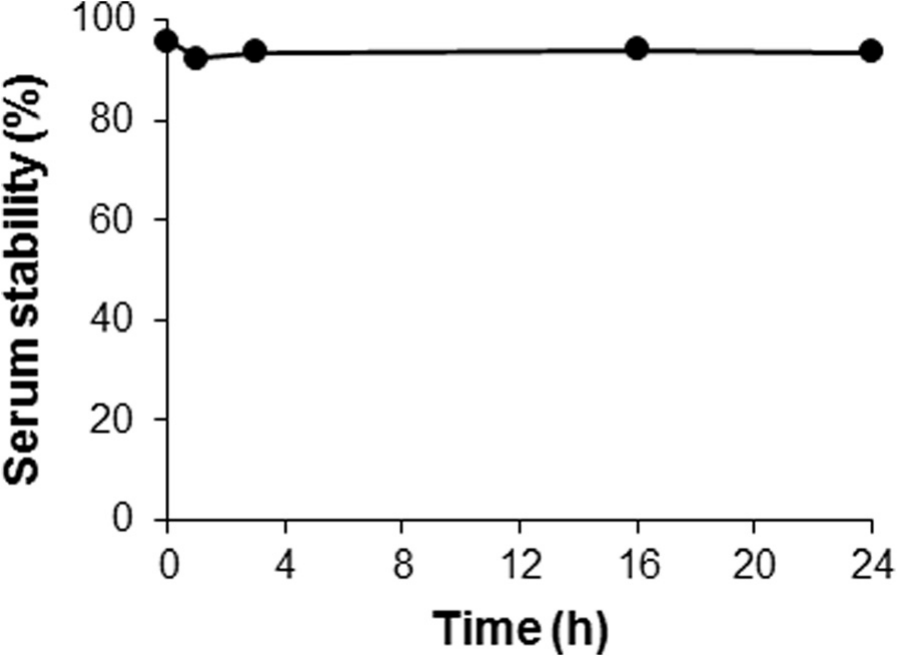
Serum stability of the ^64^Cu-DOTA-(AF)SAv/biotin-PEG-RGD_2_. Stability was measured using PD-10 columns, and percent radioactivity was determined relative to the value at 0 h (100 %)

In vitro serum stability of the ^64^Cu-DOTA-(AF)SAv/biotin-PEG-RGD_2_ was measured by incubating the hybrid probe in 50 % serum for 24 h. The results demonstrated that ^64^Cu remained stable on the hybrid probe after the 24-h incubation, showing 96.4 % radioactivity at 1 h, 97.8 % at 3 h, 98.1 % at 16 h, and 97.6 % at 24 h relative to a value of 100 % radioactivity at 0 h (Fig. 2).

### Cell integrin receptor binding

**Fig. 3:**
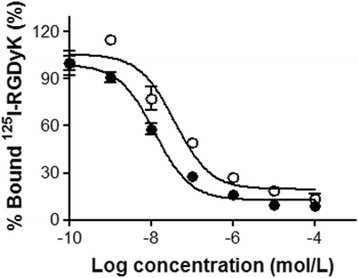
Integrin α_V_β_3_ receptor binding of the hybrid probe. ^125^I-RGDyK was used as the radioligand. IC_50_ values of DOTA-(AF)SAv/biotin-PEG-RGD_2_ (*closed circles*) and RGD_2_ (*open circles*) were 12.1 ± 4.2 nM and 37.5 ± 18.3 nM, respectively (*n* = 3)

Receptor binding affinity measurements of DOTA-(AF)SAv/biotin-PEG-RGD_2_ and RGD_2_ were performed using U87MG cells and ^125^I-RGDyK as the radioligand. IC_50_ values of DOTA-(AF)SAv/biotin-PEG-RGD_2_ and RGD_2_ were 12.1 ± 4.2 nM and 37.5 ± 18.3 nM, respectively, indicating that the former had higher binding affinity for integrin α_V_β_3_ than RGD_2_ (Fig. 3).

### Cellular uptake

**Fig. 4:**
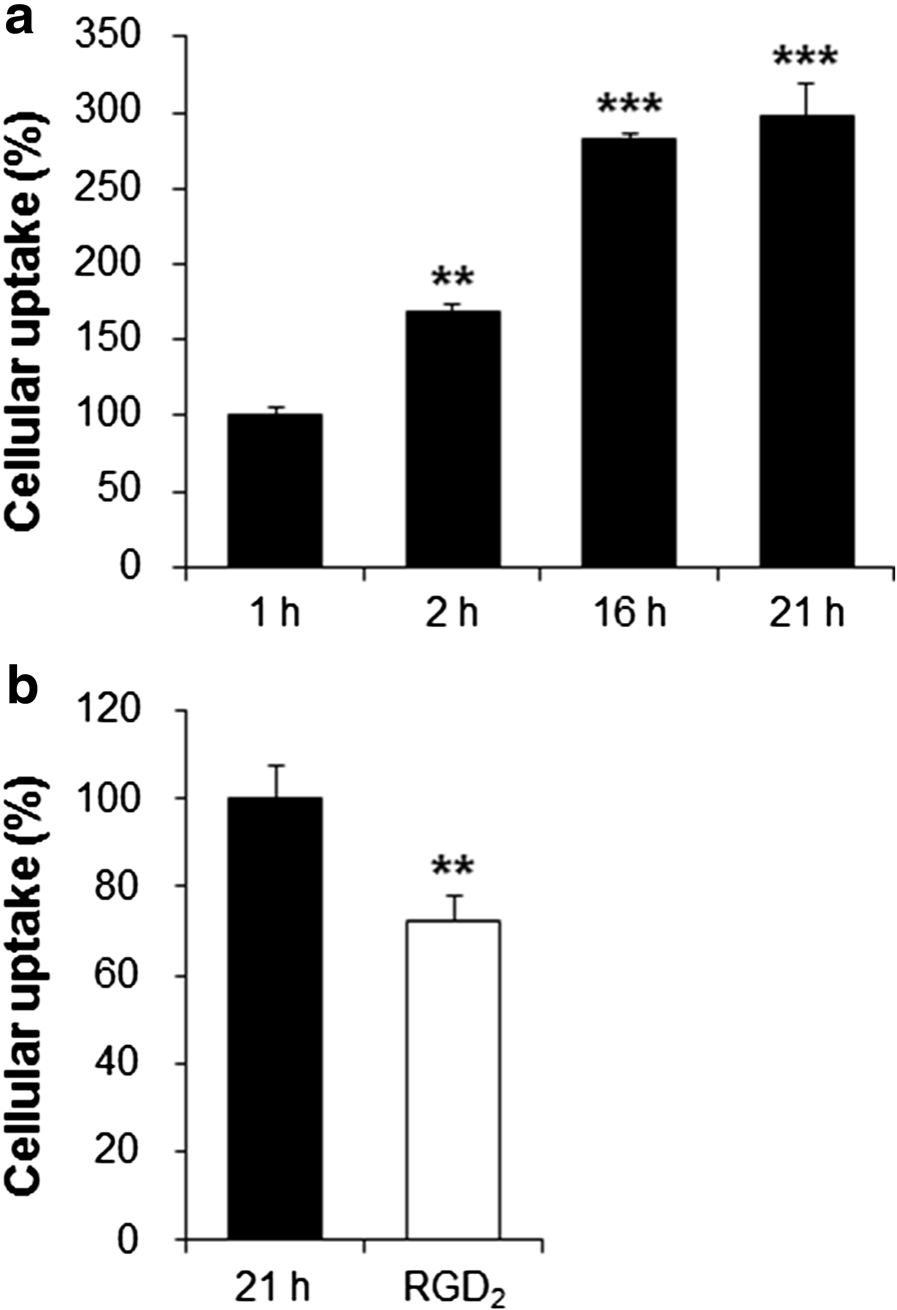
**a** U87MG cellular uptake of the ^64^Cu-DOTA-(AF)SAv/biotin-PEG-RGD_2_ as a function of time. **b** Cellular uptake of the hybrid probe at 21 h into the incubation (*black*) and after co-incubation with 10 μM of RGD_2_ (*white*). Data are means ± SDs from triplicate experiments. **P < 0.01 and ***P < 0.001

Cellular uptake of the ^64^Cu-DOTA-(AF)SAv/biotin-PEG-RGD_2_ increased in a time-dependent manner from 100.0 % at 1 h to 298.5 % at 21 h (Fig. 4a). In the blocking study, the cellular uptake decreased to 72.2 % in the presence of RGD_2_ (10 μM), indicating specific binding of the hybrid probe to the integrin α_V_β_3_ expressed on tumor cells (Fig. 4b).

### MicroPET/optical imaging

**Fig. 5:**
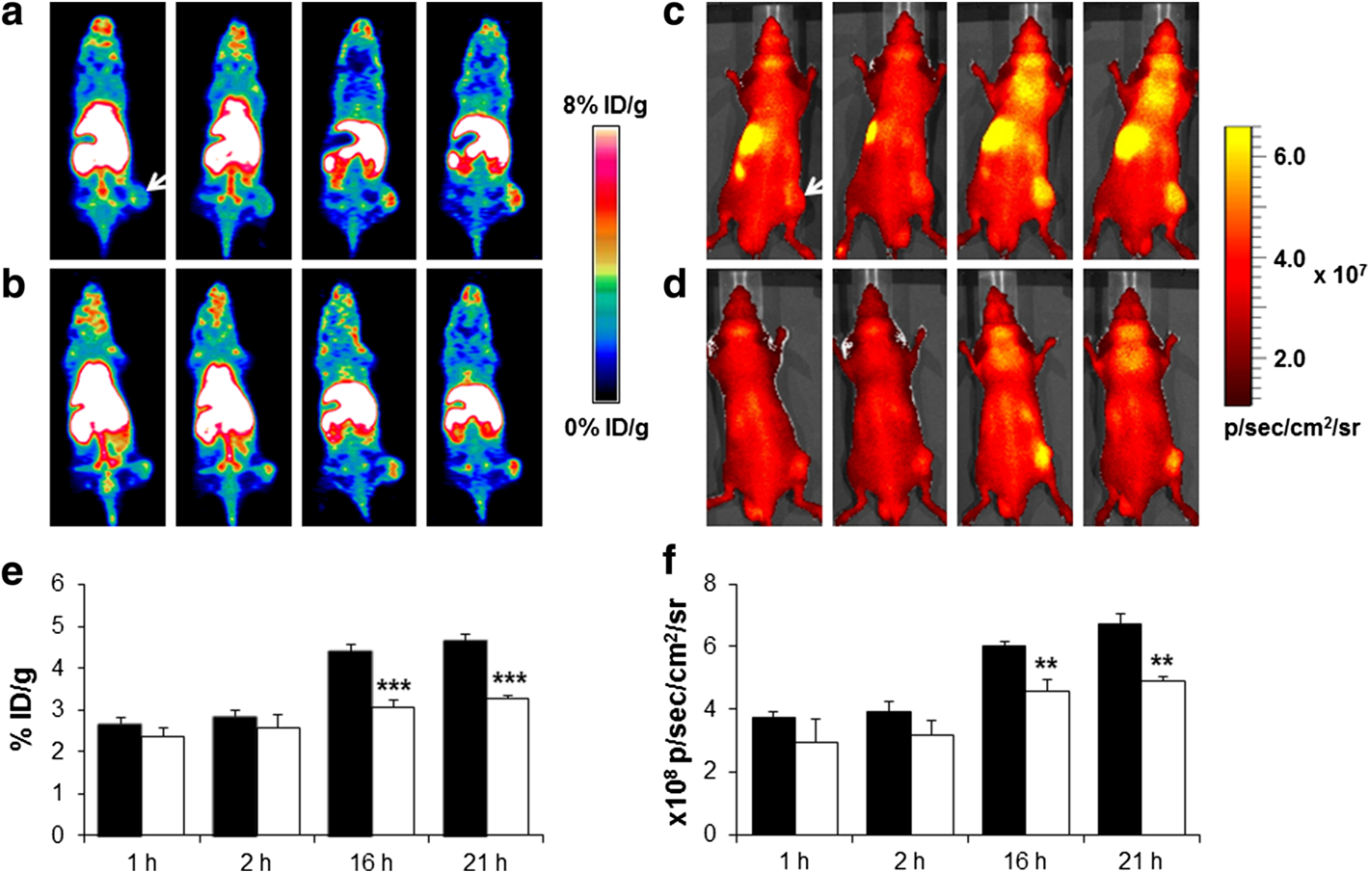
**a** MicroPET images of the ^64^Cu-DOTA-(AF)SAv/biotin-PEG-RGD_2_ in U87MG tumor-bearing mice at 1, 2, 16, and 21 h after injection and (**b**) after co-injection with RGD_2_ (20 mg/kg). **c** Optical images at the same time points and (**d**) after co-injection with RGD_2_ (20 mg/kg). *Arrows* indicate tumors. **e** ROI analysis of radioactivity uptake in tumors obtained from microPET images (**a**, **b**): control (*black*) and blocking (*white*) groups. **f** ROI analysis of fluorescence intensity in tumors obtained from optical images (**c**, **d**): control (*black*) and blocking (*white*) groups. Values represent mean % ID/g and error bars indicate SD (*n* = 3). **P < 0.01, ***P < 0.001

In vivo microPET and optical imaging of U87MG tumor-bearing mice showed high uptake of the ^64^Cu-DOTA-(AF)SAv/biotin-PEG-RGD_2_ in the liver, spleen, and tumor (Fig. 5a). ROI values of tumor tissues were 2.7 ± 0.2 % ID/g at 1 h, 2.8 ± 0.2 % ID/g at 2 h, 4.4 ± 0.2 % ID/g at 16 h, and 4.7 ± 0.2 % ID/g at 21 h (Fig. 5e). In the blocking study, tumor uptake was inhibited by 30.3 % in the presence of RGD_2_ (20 mg/kg) at 16 h after injection (Fig. 5b, e). In optical images, strong fluorescence signals were detected in the liver and tumor, which was a similar uptake pattern to that observed in the PET images (Fig. 5c, d). ROI values of tumor tissues revealed fluorescence signals of (3.7 ± 0.2) × 10^8^ photons/s/cm^2^/sr at 1 h, (3.9 ± 0.3) × 10^8^ photons/s/cm^2^/sr at 2 h, (6.0 ± 0.2) × 10^8^ photons/s/cm^2^/sr at 16 h, and (6.7 ± 0.3) × 10^8^ photons/s/cm^2^/sr at 21 h. In the presence of RGD_2_, signal intensity decreased to 4.9 ± 0.2 × 10^8^ photons/s/cm^2^/sr at 21 h (Fig. 5f).

### Ex vivo imaging and biodistribution studies

**Fig. 6:**
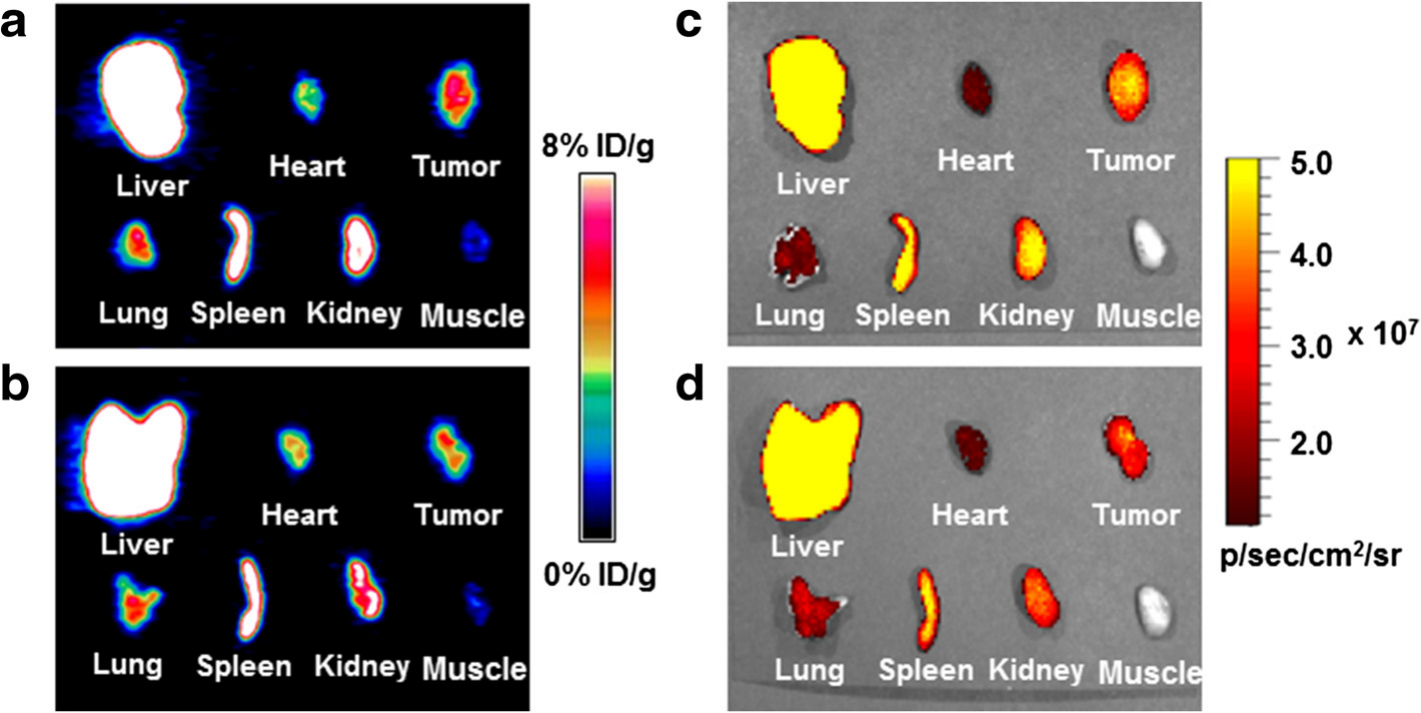
Ex vivo microPET (**a** control; **b** blocking) and optical images (**c** control; **d** blocking) of major tissues obtained after in vivo imaging (21 h)

Ex vivo PET and optical imaging carried out immediately after in vivo imaging demonstrated high signal intensity in the liver, spleen, and tumor, consistent with the in vivo imaging data (Fig. 6a–d). The ROI values of ex vivo PET images were 25.4 ± 3.1 % ID/g in the liver, 22.4 ± 1.9 % ID/g in the spleen, 5.0 ± 0.1 % ID/g in the tumor, and 0.9 ± 0.1 % ID/g in the muscle. In the blocking study, the tumor ROI value was inhibited by 34.9 %. Similarly, ROI analysis of ex vivo optical images showed (16.0 ± 1.0) × 10^7^ photons/s/cm^2^/sr in the liver, (4.2 ± 0.2) × 10^7^ photons/s/cm^2^/sr in the spleen, and (3.0 ± 0.2) × 10^7^ photons/s/cm^2^/sr in the tumor (Fig. 6c). In the blocking group, the fluorescence intensity of the hybrid probe in the tumor decreased to (2.4 ± 0.1) × 10^7^ photons/s/cm^2^/sr (Fig. 6d).

**Fig. 7:**
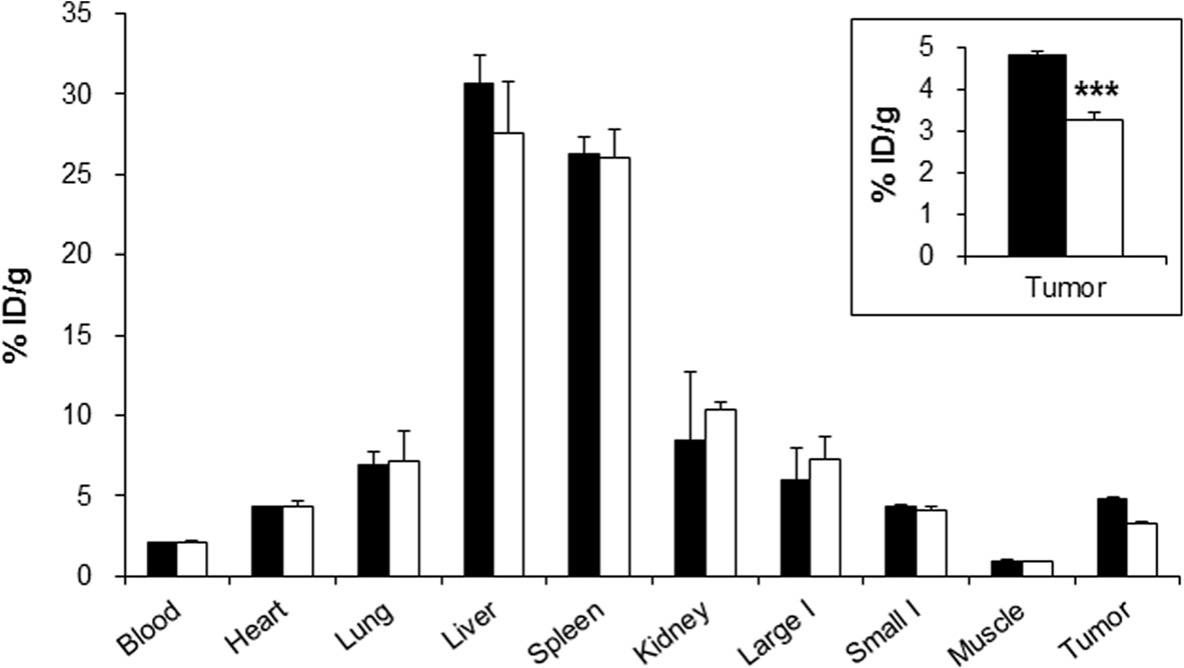
Biodistribution data of the ^64^Cu-DOTA-(AF)SAv/biotin-PEG-RGD_2_ in U87MG tumor-bearing mice at 21 h after injection (*black*) and after co-injection with RGD_2_ (*white*). *Inset* is an enlarged graph showing tumor uptake of the control (*black*) and blocking (*white*) groups. I indicates intestine. Values represent mean % ID/g and error bars indicate SD (*n* = 3). ***P < 0.001

The biodistribution study performed after ex vivo imaging confirmed the in vivo and ex vivo microPET/optical imaging data; 30.6 ± 1.7 % ID/g in the liver, 26.2 ± 1.1 % ID/g in the spleen, and 4.8 ± 0.1 % ID/g in the tumor (Fig. 7). In the blocking study using RGD_2_, tumor uptake of the hybrid probe was inhibited by 32.3 %, whereas there was no significant uptake inhibition in the blood or in other organs (Fig. 7). Tumor-to-muscle and tumor-to-blood uptake ratios obtained with the hybrid probe were 5.0 and 2.3, respectively, whereas ratios in the blocking group were 3.7 and 1.6, respectively, indicating the high specificity of the ^64^Cu-DOTA-(AF)SAv/biotin-PEG-RGD_2_ for integrin α_V_β_3_.

### Immunofluorescence staining

**Fig. 8:**
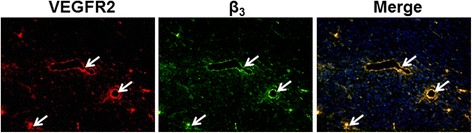
Immunofluorescence staining of U87MG tumor tissue sections obtained after microPET/optical imaging. Cell nuclei are shown as *blue dots*. Magnification ×200. *Arrows* indicate overlay areas

Immunofluorescence staining of tumor tissue sections of mice injected with the ^64^Cu-DOTA-(AF)SAv/biotin-PEG-RGD_2_ confirmed expression of β_3_ (green) and VEGFR2 (red), and the fluorescence signals from β_3_ overlapped with those of VEGFR2 (Fig. 8). This result indicates formation of new blood vessels and tumor angiogenesis in the tumor tissues.

## Discussion

DOTA-(AF)SAv and biotin-PEG-RGD_2_ were prepared via amide bond formation under basic conditions (pH 8.5). Different methods were applied to purify these two molecules due to differences in their molecular weights from reactants; DOTA-(AF)SAv was purified using a PD-10 column, in which the unconjugated DOTA was easily removed because of its low molecular weight (mw 501.49), whereas biotin-PEG-RGD_2_ was purified using a centrifugal filter to remove unreacted RGD_2_ peptide (mw 1350.43), followed by a reverse-phase HPLC column because of the small difference in molecular weight between biotin-PEG-NHS ester (mw 3790.48) and biotin-PEG-RGD_2_ (mw 5025.83). As a result, there were no molecular ion peaks corresponding to both biotin-PEG-NHS ester and RGD_2_ detected on MALDI-TOF mass spectrometry, except for the molecular ion peak of biotin-PEG-RGD_2_. The key to preparing a SAv/biotin complex is to remove unbound biotin-PEG-RGD_2_ after the complex formation. In this study, we used a spin column (mw cut-off 7 kDa) to rapidly remove molecules with a molecular weight less than 7 kDa from the ^64^Cu-DOTA-(AF)SAv/biotin-PEG-RGD_2_.

SAv/biotin complexes have previously been used to develop imaging probes; IRDye800-SAv/biotin-Avi-VEGF_121_ and ^64^Cu-DOTA-(AF)SAv/biotin-PEG-VEGF_121_ were shown to have potential for NIRF and PET/optical imaging of VEGF receptor expression, respectively [23, 26]. Moreover, SAv bound to biotinylated ^111^In-DOTA, Cy5.5, and anti-Her2 antibody showed high tumor uptake in SUMI190 tumor-bearing mice [22]. In our study, RGD_2_ was conjugated to biotin via PEG to improve its in vivo properties and provide flexibility, while ^64^Cu-DOTA was directly conjugated to SAv(AF). This imaging probe design may be suitable for hybrid imaging of tumor angiogenesis, because RGD_2_ can be used for α_V_β_3_ receptor binding while the radioisotope and fluorescent dye can be used for PET and optical imaging, respectively. A long PEG chain (mw 3400) was used to allow RGD_2_ to gain access to the binding site of the integrin receptor, while the radioisotope and fluorescent dye were conjugated directly to SAv not to interfere with binding of RGD_2_ to the integrin receptor. Furthermore, the hybrid probe was stable for 24 h based on an in vitro serum stability study (Fig. 2). In comparison with in vivo PET imaging of ^64^Cu-DOTA-(AF)SAv without biotin molecules [23], the ^64^Cu-DOTA-(AF)SAv/biotin-PEG-RGD_2_ showed higher tumor uptake that increased in a time-dependent manner, reflecting its long-term stability in vivo.

To evaluate the receptor binding affinity of the probe, U87MG cells, which are known to express high levels of integrin α_V_β_3_ [27], were incubated with ^125^I-RGDyK in the presence of different concentrations of DOTA-(AF)SAv/biotin-PEG-RGD_2_ or RGD_2_. IC_50_ value of DOTA-(AF)SAv/biotin-PEG-RGD_2_ was 3.1-fold higher than that of RGD_2_ (Fig. 3). Diverse dimeric and multimeric RGD peptides have been shown to have higher in vitro receptor binding affinity and tumor uptake than monomeric RGD peptides [12, 28]. In this study, four equivalents of the RGD_2_ peptide were possibly bound to one molecule of SAv because of its tetrameric structure, which might have contributed to the higher binding affinity of the ^64^Cu-DOTA-(AF)SAv/biotin-PEG-RGD_2_ than the dimeric RGD peptide probably due to a polyvalency effect [9, 10]. Cellular uptake study exhibited that the hybrid probe was taken up by U87MG cells in a time-dependent manner, and that its levels increased 3.0-fold over a 21-h incubation (Fig. 4a). Cellular uptake was inhibited significantly by RGD_2_, indicating specificity of the probe to the integrin α_V_β_3_ receptor (Fig. 4b).

In vivo microPET and optical imaging results of ^64^Cu-DOTA-(AF)SAv/biotin-PEG-RGD_2_ demonstrated incremental tumor uptake over time. ROI values of tumor uptake obtained from microPET images increased 1.7-fold over 21 h (Fig. 5a). A similar pattern of tumor uptake was observed in optical images, with uptake increasing 1.8-fold over the same period of time (Fig. 5c). Biodistribution data of this hybrid probe demonstrated higher tumor uptake (4.8 ± 0.1 % ID/g at 21 h after injection) than that of ^64^Cu-DOTA-E[(RGDfK)]_2_ in U87MG tumor-bearing mice (<4 % ID/g at 4 h after injection) [9], although ^64^Cu-DOTA-E[(RGDyK)]_2_ showed better in vivo kinetics than the D-Phe derivative in MDA-MB-435 tumor-bearing mice [10]. Furthermore, the tumor uptake pattern of ^64^Cu-DOTA-(AF)SAv/biotin-PEG-RGD_2_ was similar to that reported for other RGD-conjugated nanoparticles. ^64^Cu-DOTA-QDot-RGD showed relatively low tumor uptake at 1 h after injection (less than 1 % ID/g), which then increased to 4.3 ± 0.5 % ID/g at 25 h after injection based on ROI analysis of microPET images [16]. Higher tumor uptake was detected in ^64^Cu-labeled RGD-conjugated iron oxide nanoparticles, which were avidly taken up by U87MG tumors of mice (7.9 ± 0.8 % ID/g at 1 h after injection) and increased to 9.8 ± 3.2 % ID/g at 21 h after injection [17]. Another study showed that ^64^Cu-DOTA- and RGD-conjugated single-walled carbon nanotubes exhibited significantly high tumor uptake of 10–15 % ID/g at 24 h after injection compared to 3–4 % ID/g uptake of RGD-free nanotubes [19]. The presence of SAv in the hybrid probe may affect tumor uptake, because ^111^In-labeled SAv was reported to accumulate in tumors in LS174T tumor mice (4.5 ± 0.2 % ID/g at 5 h after injection) [29]. In our previous study, however, ^64^Cu-DOTA-(AF)SAv without biotin molecules showed low tumor uptake (1.6 ± 0.1 % ID/g at 22 h) [23]. Therefore, tumor uptake of the ^64^Cu-DOTA-(AF)SAv/biotin-PEG-RGD_2_ was likely due to the probe itself.

The hybrid probe showed high uptake in the liver and spleen compared with that of ^64^Cu-DOTA-E[(RGDfK)]_2_ [9]. This high uptake in the reticuloendothelial system resulted from the presence of the relatively large molecule, SAv, as shown by high liver uptake of ^64^Cu-DOTA-(AF)SAv (15.7 ± 0.57 % ID/g at 22 h) [23]. A similar uptake pattern was detected in microPET images of U87MG tumor-bearing mice injected with ^64^Cu-DOTA-QDot-RGD; liver uptake was higher than 40 % ID/g up to 25 h after injection based on ROI analysis [16]. ^64^Cu-labeled RGD-conjugated iron oxide nanoparticles also showed high liver uptake (31.1 ± 2.5 % ID/g at 1 h and 11.7 ± 1.2 % ID/g at 21 h after injection), probably due to the relatively large size of the particles [17]. Similarly, high liver uptake (17~30 % ID/g at 24 h) was also detected in mice injected with ^64^Cu-labeled single-walled carbon nanotubes coated with phospholipid-PEG chains conjugated to the RGD peptide [19]. The longer the PEG chain, the lower the liver uptake and the higher the tumor uptake of nanotubes [19]. Therefore, use of longer PEG arms may improve the in vivo properties of the ^64^Cu-DOTA-(AF)SAv/biotin-PEG-RGD_2_ by reducing liver uptake. Optical images confirmed the microPET imaging data for the hybrid probe and showed high fluorescence intensity in tumors that increased over time (Fig. 5c, d). While the ^64^Cu-DOTA-(AF)SAv/biotin-PEG-RGD_2_ was taken up by the liver and spleen based on PET imaging, this was not clear from optical images due to the presence of the skeleton and the depth penetration limitations of optical imaging. However, ex vivo optical images revealed that the hybrid probe was taken up by the liver, spleen, and tumor (Fig. 6c, d).

Immunofluorescence staining of tumor tissue sections obtained after microPET/optical imaging demonstrated that β_3_ receptors were highly expressed on endothelial cells (VEGFR2) of blood vessels (Fig. 8). This result suggests that tumor uptake of the hybrid probe was attributable to its binding to integrin α_V_β_3_.

Our study showed that both microPET and optical imaging data of ^64^Cu-DOTA-(AF)SAv/biotin-PEG-RGD_2_ in U87MG tumor-bearing mice were in good agreement for monitoring integrin α_V_β_3_ expression. This hybrid probe provided real-time detection of tumor as well as quantitative information on tumor and major organs with high specificity and sensitivity. In addition, this hybrid probe may have advantages, because hybrid PET/optical probes may allow for preoperative detection, intraoperative image-guided surgery, and postoperative evaluation of tumors.

## Conclusions

We prepared a ^64^Cu-DOTA-(AF)SAv/biotin-PEG-RGD_2_ efficiently using a SAv/biotin complex as a platform. As shown by a receptor binding study, DOTA-(AF)SAv/biotin-PEG-RGD_2_ exhibited higher binding affinity for the integrin α_V_β_3_ than RGD_2_ peptide, which we attributed to a possible polyvalency effect by four flexible biotin-PEG-RGD_2_ arms complexed with one SAv core. PET/optical images of U87MG tumor-bearing mice indicated that the hybrid probe accumulated in tumor with prolonged retention over 21 h. These results demonstrate that ^64^Cu-DOTA-(AF)SAv/biotin-PEG-RGD_2_ provides specific and sensitive hybrid PET/optical images for monitoring integrin α_V_β_3_ expression, and that a SAv/biotin complex is a useful platform for developing hybrid imaging probes.
